# Working Memory Training for Children Using the Adaptive, Self-Select, and Stepwise Approaches to Setting the Difficulty Level of Training Activities: Protocol for a Randomized Controlled Trial

**DOI:** 10.2196/47496

**Published:** 2023-09-19

**Authors:** Regine Cassandra Lau, Peter John Anderson, Joshua F Wiley, Derek Huang, Faisha Surjatin, Paul McIntosh, Susan Gathercole, Megan Spencer-Smith

**Affiliations:** 1 School of Psychological Sciences and Turner Institute for Brain and Mental Health Monash University Clayton Australia; 2 Victorian Infant Brain Studies (VIBeS) Clinical Sciences Murdoch Children’s Research Institute Melbourne Australia; 3 Peter MacCallum Cancer Centre Melbourne Australia; 4 Virtual and Augmented Reality Services (VARS) eSolutions Monash University Clayton Australia; 5 MRC Cognition and Brain Sciences Unit University of Cambridge Cambridge United Kingdom

**Keywords:** children, working memory, memory training, adaptive training, cognitive training, transfer effects, training effects, cognitive outcomes, randomized controlled trial

## Abstract

**Background:**

A common yet untested assumption of cognitive training in children is that activities should be adaptive, with difficulty adjusted to the individual’s performance in order to maximize improvements on untrained tasks (known as transfer). Working memory training provides the ideal testbed to systematically examine this assumption as it is one of the most widely studied domains in the cognitive training literature, and is critical for children’s learning, including following instructions and reasoning.

**Objective:**

This trial aimed to examine children’s outcomes of working memory training using adaptive, self-select (child selects difficulty level), and stepwise (difficulty level increases incrementally) approaches to setting the difficulty of training activities compared to an active control condition immediately and 6-month postintervention. While the aim is exploratory, we hypothesized that children allocated to a working memory training condition would show greater improvements: (1) on near transfer measures compared to intermediate and far transfer measures and (2) immediately postintervention compared to 6-month postintervention.

**Methods:**

This double-blinded, active-controlled, parallel-group randomized trial aimed to recruit 128 children aged 7 to 11 years from 1 metropolitan primary school in Melbourne, Australia. Following baseline testing, children were randomized into 1 of 4 conditions: adaptive, self-select, or stepwise working memory training, or active control. An experimental intervention embedded in Minecraft was developed for teachers to deliver in class over 2 consecutive weeks (10 × 20-minute sessions). The working memory training comprised 2 training activities with processing demands similar to daily activities: backward span and following instructions. The control comprised creative activities. Pre- and postintervention, children completed a set of working memory tests (near and intermediate transfer) and the Raven’s Standard Progressive Matrices (far transfer) to determine training outcomes, as well as motivation questionnaires to determine if motivations toward learning and the intervention were similar across conditions. Caregivers completed the ADHD-Rating Scale-5 to measure their child’s attention (far transfer). Statistical analysis will include traditional null hypothesis significance testing and Bayesian methods to quantify evidence for both the null and alternative hypotheses.

**Results:**

Data collection concluded in December 2022. Data are currently being processed and analyzed.

**Conclusions:**

This trial will determine whether the adaptive approach to setting the difficulty of training activities maximizes cognitive training outcomes for children. This trial has several strengths: it adopts best practices for cognitive training studies (design, methods, and analysis plan); uses a range of measures to detect discrete levels of transfer; has a 6-month postintervention assessment; is appropriately powered; and uses an experimental working memory training intervention based on our current understanding of the cognitive mechanisms of training. Findings will inform future research and design of cognitive training interventions and highlight the value of the evidence-based principles of cognitive training.

**Trial Registration:**

Australian New Zealand Clinical Trials Registry, ACTRN12621000990820; https://www.anzctr.org.au/ACTRN12621000990820.aspx

**International Registered Report Identifier (IRRID):**

DERR1-10.2196/47496

## Introduction

### Background

Cognitive training interventions targeting core cognitive abilities such as working memory, attention, and problem-solving are in demand. Supporters of cognitive training explain that repeated practice on an activity will lead to performance gains that can transfer to improved performance on untrained tasks, an outcome known as transfer [1-4]. Transfer is the ultimate goal of cognitive training, and this intervention method is therefore of considerable interest to practitioners in health and education sectors seeking to prevent and ameliorate the disadvantage associated with low cognitive abilities, including inattention and learning related challenges (eg, psychologists, psychiatrists, and other mental health professionals; speech and language therapists; pediatricians; and teachers, special education needs coordinators, and specialist teachers). Transfer has been described on a continuum, ranging from near transfer to far transfer*. Near transfer* refers to improvement on measures structurally similar to the training activity, for example, performance on a measure using the same paradigm as the training activity but different stimulus, mode of presentation, or response modality. *Far transfer* focuses on performances on measures beyond the trained cognitive domain that rely on the trained skill. More recently, researchers have also been examining *intermediate transfer*, which describes improvement on measures that assess the same cognitive domain but use a different paradigm to the training activity. Working memory training benefits typically diminish with time, and with decreasing similarities between the training activity and outcome measure, providing consistent evidence for near transfer, less evidence for intermediate transfer, and little convincing evidence for far transfer [5-8].

A key design feature of cognitive training that is assumed to maximize training outcomes is *adaptivity* of the training activities. In this approach, the difficulty of the training activity is adjusted to the trainee’s performance, thought to sufficiently challenge the trainee’s cognitive limits to induce plasticity [9-12]. Indeed, most commercially available cognitive training programs are adaptive. Initial support for adaptive cognitive training in children came from studies demonstrating greater training effects and transfer following adaptive training, in which training difficulty varied, compared to a nonadaptive control, in which training difficulty was fixed at a low level [11,13-16]. This design is problematic for evaluating the adaptive method, with adaptivity and difficulty of the training activities confounded: children in the adaptive condition were exposed to varying difficulty levels, but those in the nonadaptive condition only experienced a single difficulty level. To address this important limitation, von Bastian and Eschen [7] systematically examined in adults the outcomes of working memory training with difficulty of the training activities being adaptive, self-selected, or randomized compared to an active control. The adults in the adaptive condition outperformed those in the active control condition on the training activity, but this was also the finding for the self-selected and randomized conditions. While near transfer effects were not examined, none of the training conditions showed improvements on intermediate or far transfer tests. The authors postulated that varying the difficulty level of the training activities may be sufficient to induce training effects, and that training and transfer effects may not be modulated by a specific approach to setting the difficulty of a training activity. These findings question the common assumption that an adaptive approach is superior for maximizing cognitive training outcomes.

Working memory training provides the ideal testbed to systematically examine the assumption that the adaptive approach maximizes cognitive training outcomes. It is one of the most widely studied domains in the cognitive training literature [17,18]. Working memory is a mental workspace capable of holding and processing information for brief periods in the course of ongoing cognitive activity [19]. This ability is critical for children’s learning as it facilitates a host of complex cognitive activities such as comprehension, reasoning, and problem-solving [20]. Indeed, low working memory is commonly associated with poor scholastic attainments [20,21]. Meta-analyses examining near transfer effects following cognitive training reveal the highest effect sizes for training targeting working memory (*g*=0.50) than either inhibitory control (*g*=0.24) or cognitive flexibility (*g*=0.37) [6,17]. Evidence from high-quality trials also demonstrates near transfer effects of working memory training programs in children, such as Cogmed [15,22] and Braintwister [16,23]. However, there is less support for intermediate transfer, and the evidence for far transfer is negligible when methodological issues, such as the lack of an active control group, are taken into account [6,8,17,22,24-26].

### Aims and Hypotheses

This randomized controlled trial assessed the common assumption that cognitive training in children should be adaptive. The trial aimed to examine children’s outcomes of working memory training using adaptive, self-select, and stepwise approaches to setting the the difficulty of training activities compared to an active control condition immediately and 6-month postintervention. Although the aim is exploratory, we hypothesized that children allocated to the working memory training conditions would show greater improvements: (1) on near transfer compared to intermediate and far transfer measures and (2) immediately postintervention compared to 6-month postintervention.

## Methods

### Ethics Approval

This trial was approved by the Monash University Human Research Ethics Committee (24305) in January 2021 and Melbourne Archdiocese Catholic Schools (1066) in February 2021. It was registered with the Australian New Zealand Clinical Trials Registry (ACTRN12621000990820) on July 28, 2021. We note that due to time constraints associated with COVID-19 lockdowns in Melbourne, Australia, the first participant was enrolled in the trial on July 13, 2021, when approval from the Australian New Zealand Clinical Trials Registry was pending, resulting in the trial’s retrospective registration status.

### Design and Blinding

**Figure 1:**
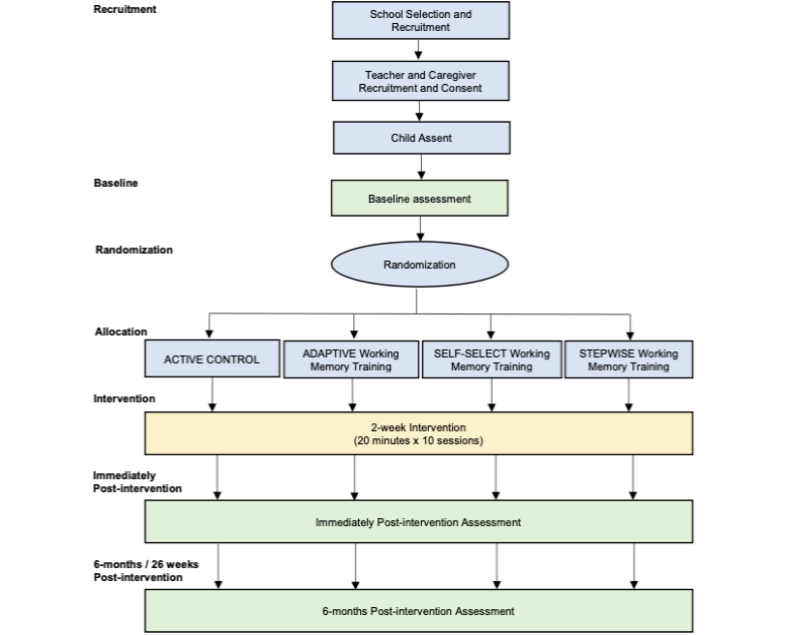
Trial design.

This double-blinded, active-controlled, parallel-group, randomized superiority trial is part of the larger Common Assumptions of Cognitive Training (COMET) study. Researchers involved in child assessments were blinded to the child’s intervention allocation and past assessment results. Teachers, caregivers, and children were informed that the study was examining children’s thinking skills, and they were unaware of the different conditions. Thus, participants were unaware that this was a randomized controlled trial with different conditions, thereby preserving blinding. This trial was conducted and will be reported according to CONSORT (Consolidated Standards of Reporting Trials) guidelines. Figure 1 summarizes the trial design. This trial was expected to run for 15 months, with school selection and recruitment taking 3 months, and consent, data collection, and intervention carried out over 12 months.

### Setting

The research was conducted in a primary school in metropolitan Melbourne in the state of Victoria (population of 6.6 million in 2022), Australia [27].

### School Recruitment

Eligible primary schools were within 20 km of the Monash University Clayton campus (for practical reasons) and had at least 197 children in grades 2 to 5 (to achieve the required sample size based on power calculations). Schools were randomly invited until one agreed to participate.

### Participant Recruitment

All children in grades 2 to 5 (7 to 11 years) at the participating school were invited to participate in this trial. The grade 2 to 5 teachers distributed the caregiver explanatory statement and caregiver consent form for the children to take home to their caregivers. Caregivers were asked to provide consent for completing questionnaires about themselves and their child, and for their child to participate in cognitive testing. The children returned the completed consent forms to their teachers to pass on to the research team. Child assent was obtained during the baseline child testing session. Consent was sought from the teachers for their involvement in obtaining caregiver consent.

### Eligibility Criteria

Children were included if they had caregiver consent, and provided written assent to participate. They were excluded if they had caregiver-reported vision impairment that cannot be corrected by glasses, including color vision deficiency, hearing impairment that cannot be corrected by a hearing aid, fine motor impairment, or intellectual disability that would prevent participation in the intervention.

### Randomization

Following baseline testing, children were randomized to 1 of the 4 number-coded conditions (adaptive, self-select, stepwise working memory training, or active control) using a randomization scheme generated in advance and set up in Research Electronic Data Capture (REDCap) hosted at Monash University by the trial statistician (JFW) who was not involved in data collection. Specifically, block sizes of variable size (4 or 8) were used to assure allocation concealment and preguessing of the allocation sequence at the end of each block. Randomization was stratified by age at baseline: 7 to 8 years, 9 to 10 years, and 11 years.

### Intervention

#### Overview

The Brain Space Program intervention was designed and developed by our team in Minecraft Education (Mojang Studios and Xbox Game Studios) for the COMET project (not an official Minecraft product, not approved by or associated with Mojang), and in this trial includes 3 working memory training conditions (adaptive, self-select, and stepwise) and an active control condition. All conditions were in the same Minecraft environment and had the same motivating features, including a “space mission” narrative for each session and the acquisition of experience points that could be used at the end of each training session for creative activities.

The teacher delivered the intervention in class. Children completed a session lasting 20 minutes on each school day for 2 consecutive weeks (total 10 sessions, total dose 200 minutes). Children individually performed the intervention on an iPad with headphones (provided by the researchers) to reduce distraction. To account for missed sessions (eg, child absences, school events, and public holidays), children were allowed to complete up to 3 training sessions per day during the intervention period. Researchers were in the classroom during the intervention period to ensure protocol adherence and compliance.

#### Working Memory Training Conditions

**Figure 2:**
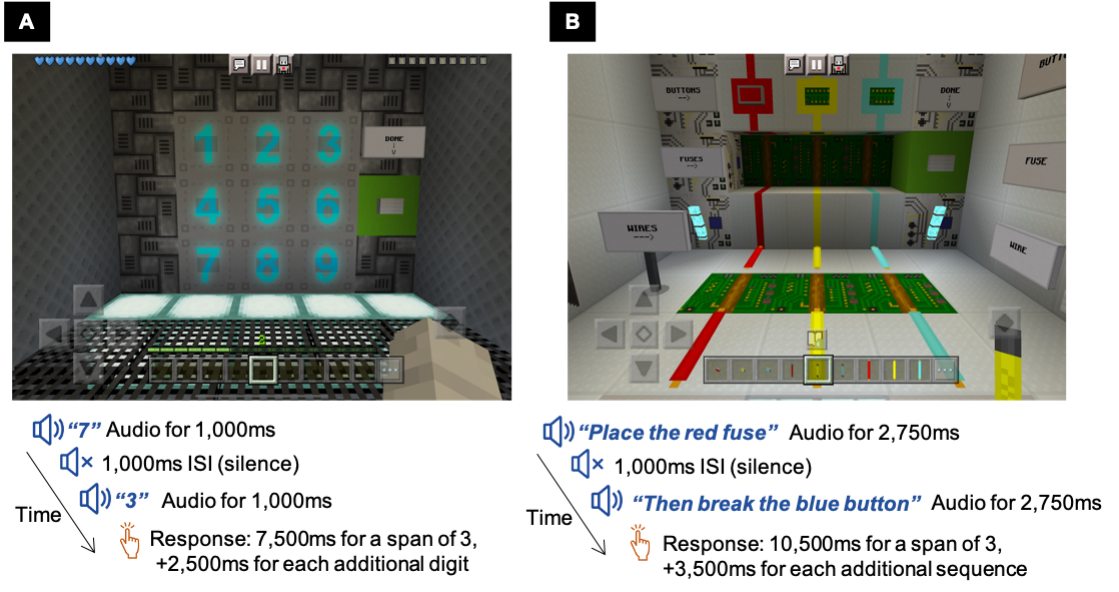
The working memory training activities: (A) backward span and (B) following instructions. ms = milliseconds, ISI = Interstimulus Interval.

The working memory training consisted of 2 activities that required the child to temporarily store and manipulate verbally presented information: a backward span activity and a following instructions activity (see Figure 2). These paradigms were selected based on the current understanding of the cognitive mechanisms of training. Importantly, first, processing demands are sufficiently unfamiliar so the child is required to generate novel cognitive routines (strategies) that improve efficiency in performance and can be applied to new activities with sufficient overlap in task structure [5,6]. Second, processing demands overlap with daily activities, such as following teacher directions, leading to potential practical benefits [2,28].

In the backward span training activity, a series of digits were presented auditorily, and the child had to remember and immediately recall in reverse order the sequence of items by tapping the digits on the iPad screen. The difficulty level was manipulated by increasing the number of digits to be recalled. The following instructions training activity is based on a classroom analog test of working memory developed by Gathercole et al [29]. In this training activity, the child was introduced to 3 objects, each object comes in 3 colors, and 2 actions that require different tapping responses on the iPad screen. The child had to remember and immediately implement an action-color-object sequence (eg, break the red wire, then place the blue button, reflecting a span of 2) by using the relevant tapping response on the screen of the iPad with the corresponding color-objects. Difficulty level was manipulated by increasing the number of action-color-objects in the sequence the child has to perform. Each activity comprised 5 blocks of trials per session, and each block had 4 trials of a span level (per session: total 20 backward span trials, then 20 following instructions trials).

In this trial, there were three different working memory training conditions, each with a different approach to adjusting the difficulty level of the training activities: (1) *adaptive* condition, the difficulty level adapted to the child’s performance during each training session on a block-by-block basis for the first 4 blocks (adapted between block 1 to 2, block 2 to 3, and block 3 to 4, and block 5 retained the same difficulty as block 4) within the training sessions for each activity; (2) *self-select* condition, the child decided at the end of each session if they would like a training activity to be easier, the same, or more difficult in the next session; and (3) *stepwise* condition, the difficulty level increased incrementally across sessions and this progression was predetermined and stratified by age based on current understanding of the development of children’s working memory: 7 years, 8 to 9 years, and 10 to 11 years [30].

#### Active Control Condition

The active control involved the child completing a series of creative building and discovery activities each session. The activities were embedded in the same environment as the working memory training activities, and therefore the condition can be considered a placebo [1]. The activities were selected based on a review of activities available on the Minecraft Education website for school teachers to use with children aged 7 to 11 years in the classroom that do not require working memory [31].

### Measures

#### Overview

Table 1 summarizes the measures, respondents, administration time points, as well as the primary outcomes of this trial.

**Table 1:** Trial measures.

Domain or measure			Respondent		Baseline		Immediately postintervention		6 months postintervention
**Child characteristics**									
	Participant information questionnaire	Caregiver		✓		N/A^a^		N/A	
**Near transfer**									
	Backward span digits	Child		✓		Primary outcome		✓	
	Following instructions objects	Child		✓		Primary outcome		✓	
	Backward span letters	Child		✓		✓		✓	
	Following instructions letters	Child		✓		✓		✓	
**Intermediate transfer**									
	2-back objects	Child		✓		✓		✓	
**Far transfer**									
	Raven’s SPM^b^ sets A to E	Child		✓		N/A		N/A	
	Raven’s SPM sets A and B	Child		N/A		✓		✓	
	ADHD-5-RS^c^	Caregiver		✓		✓		✓	
**Child motivation**									
	Intrinsic Motivation Scale	Child		✓		N/A		N/A	
	Intrinsic Motivation Inventory	Child		N/A		✓		N/A	

^a^N/A: Not applicable, test not performed at this time point.

^b^SPM: Standard Progressive Matrices.

^c^ADHD-5-RS: ADHD-Rating Scale-5, home version.

#### Child Characteristics

Caregivers completed a participant information questionnaire that collected demographic information about their child, including their child’s sex, developmental and medical history, and family socioeconomic risk factors [32].

#### Training Outcomes

##### Near Transfer

**Figure 3:**
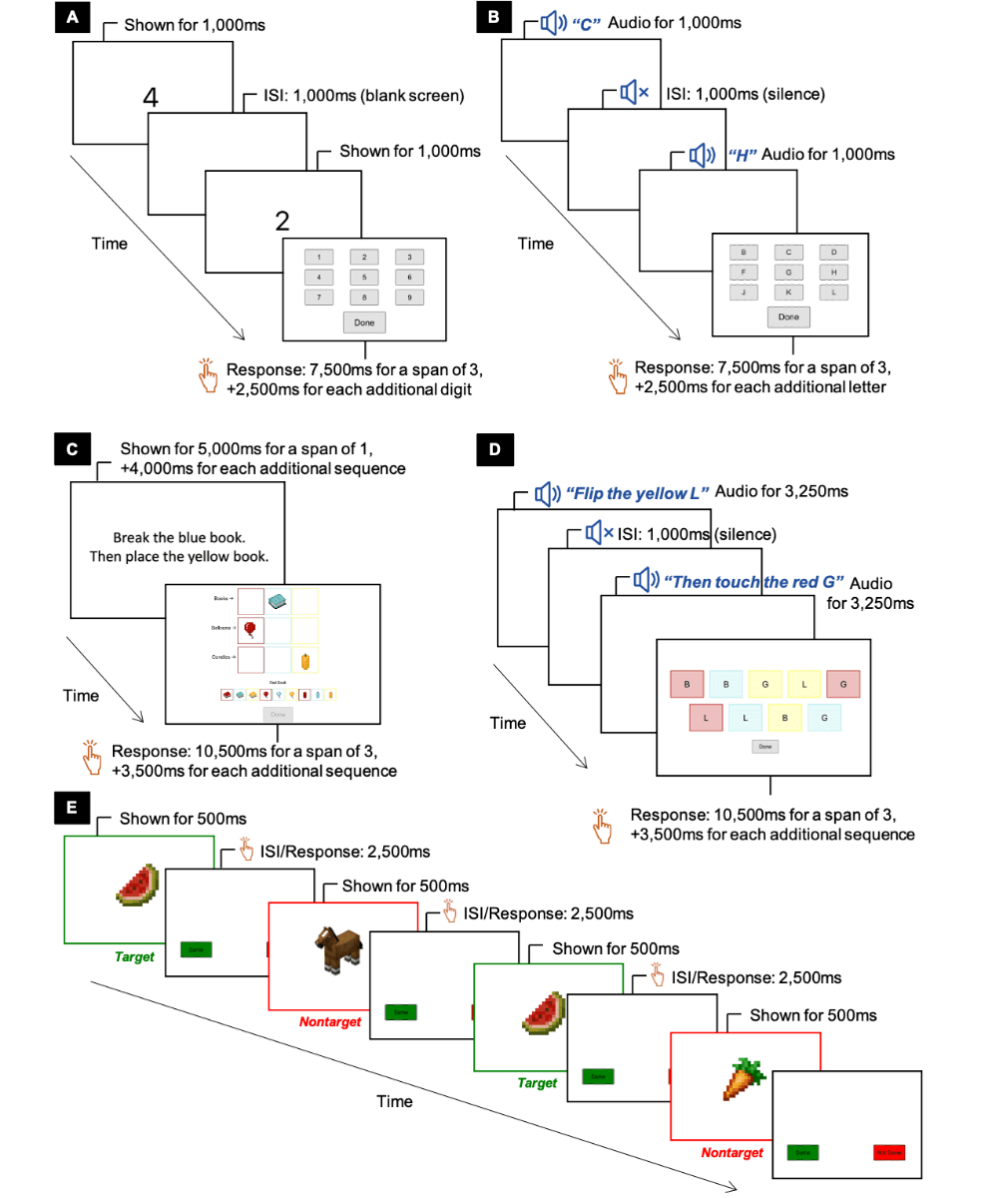
The experimental working memory transfer tests delivered on an iPad: (A) backward span digits, (B) backward span letters, (C) following instructions objects, (D) following instructions letters, (E) 2-back objects. (A) to (D) depict each test at a span level of 2. ms = milliseconds, ISI = Interstimulus Interval.

A set of four experimental working memory tests that have the same paradigms as the training activities, but different stimulus features, provided measures of near transfer (Table 2, Figures 3A to 3D): (1) *backward span* tests, digits and letters versions, and (2) *following instructions* tests, objects and letters versions [6]. For each test, there were 4 trials per block of a span level. Responses were self-paced with a cut-off duration for response time adjusted per span level. The span level increased by 1 if the child scored 3 or more out of the 4 trials in that block (≥75% accuracy); otherwise, the test ended. The tests, including instructions, practice items, and corrective feedback, were delivered via an iPad-based web app developed for this research. These multiple near transfer tests provide the opportunity to detect discrete levels of transfer by minimizing confounders associated with training effects [4].

**Table 2:** Comparison of the working memory training activities and near transfer tests. Responses provided on an iPad by tapping on-screen buttons.

Activity or test			Stimulus type	Stimulus, presentation		Response modality (response layout)
**Backward span**						
	Training activity	Digits		Auditory	Motor (3×3 keypad)	
	Near transfer test	Digits		Visual	Motor (3×3 keypad)	
	Near transfer test	Letters		Auditory	Motor (3×3 keypad)	
**Following instructions**						
	Training activity	Items: space objects; actions: place, break		Auditory	Motor (3×3 grid)	
	Near transfer test	Items: common everyday objects and animals; actions: place, break		Visual	Motor (3×3 grid)	
	Near transfer test	Items: letter cards; actions: touch, flip		Auditory	Motor (2-row keyboard)	

##### Intermediate Transfer

An experimental n-back test measured intermediate transfer (Figure 3E). In this *2-back* test, the child was presented with a continuous stream of 22 common objects and animals (eg, carrot, flower, and cat; 6 targets and 16 nontargets), and the child had to decide for each object if it matched the object that was presented 2 items ago [5,33,34]. The child responded by tapping the “same” or “not same” on-screen button within the time limit. This test was delivered via the same iPad-based web app as the near transfer tests.

##### Far Transfer

Far transfer measures assessed children’s reasoning and attention. Children’s reasoning ability was measured using the Raven’s Standard Progressive Matrices (SPM), standardized for individuals aged 6.5 to 80 years [35,36]. The Raven’s SPM contains 5 sets (A to E) of 12 items. Each item has a geometric design with a missing piece on the bottom right. The child had to select the correct figure that would complete the overall geometric design vertically and horizontally from 6 or 8 possible options. The items increased in difficulty within sets and across sets. Children were allowed as much time as they needed to complete the task. The Raven’s SPM has excellent split half-reliability and test-retest reliability (>0.80), and its concurrent validity is well established [37]. There is a moderately strong correlation (0.50 to 0.70) between SPM and conventional intelligence tests such as Stanford-Binet and Wechsler’s tests [38].

Children’s inattention and hyperactivity-impulsivity behaviors were assessed using the caregiver-rated ADHD-Rating Scale-5 (ADHD-RS-5, home version), standardized for children and adolescents aged 5 to 17 years [39]. The ADHD-RS-5 has an inattention subscale (9 items) and a hyperactivity-impulsivity subscale (9 items) that closely follow the Diagnostic and Statistical Manual of Mental Disorders (Fifth Edition) criteria for attention-deficit/hyperactivity disorder. Caregivers rated their child’s behaviors at home based on the previous 6 months, and at the immediate postintervention time point based on the past 2 weeks to assess any changes observed during the intervention period. Internal consistency of the symptom subscales (0.61 to 0.83) and concurrent validity were established by high correlations (>0.78) with respective subscales from the Connors Parent Rating Scale [39].

#### Child Motivation

##### Overview

Intrinsic motivation, an internal drive to seek out new and challenging experiences to learn and expand one’s abilities [40], has been associated with performances on cognitive training activities and outcomes in children [41-43] and adults [44]. We measured aspects of children’s motivation to rule out the potential influence of differences in expectations between the working memory training conditions and the active control [1,45].

##### Motivation Toward Classroom Activities

Children’s motivation toward typical classroom activities was measured using the Intrinsic Motivation Scale (IMS) [46]. The IMS consists of 3 subscales: challenge (6 items; eg, I like hard work because it’s difficult), curiosity (6 items; eg, I ask questions in class because I want to learn new things), and independent mastery (5 items; eg, I like to try to figure things out at school on my own). The child responded using a 3-point Likert scale (yes: 3 points; sometimes: 2 points; no: 1 point). The IMS has very high internal consistency (0.90) and high test-retest reliability with a 6-week interval (0.74) [46].

##### Motivation Toward the Intervention

Children’s motivations toward the intervention were measured using a modified Intrinsic Motivation Inventory (IMI) [47], which has been used in child cognitive training studies [41-43]. This modified IMI consists of 4 subscales: interest or enjoyment (7 items; eg, it was fun to do), perceived competence (6 items; eg, I think I am pretty good at the training), effort or importance (5 items; eg, I put a lot of effort into it), and value or usefulness (4 items; eg, I think the training could help me). Identical to the IMS, the child responded on a 3-point Likert scale. This modified IMI has been used in a previous randomized control trial of working memory training with similar-aged children and has high internal consistency (≥0.74) [41].

### Data Management and Confidentiality

Personal and trial data were collected and managed using REDCap electronic data capture tools hosted and managed by Helix (Monash University) [48,49]. Personal data required to disseminate the caregiver questionnaires were held securely and will not be used for any other purpose. Given the sample size, the trial’s timeframe, and the minimal risks associated with participation, there is no data monitoring committee.

### Procedures

Children participated in testing sessions, and caregivers completed behavioral questionnaires at baseline, immediately, and 6 months postintervention. The baseline and immediately postintervention measures were completed 1 week before and after the intervention, respectively. The 6-month postintervention measures were completed approximately 26 weeks postintervention (±8 weeks due to school term breaks).

Children were tested in small groups of 6 to 8. The group child testing sessions were led by a doctoral provisional psychologist and conducted in a quiet room on the primary school campus. The test order and administration protocol was the same at each time point. Children first completed the working memory measures on an iPad with headphones to reduce distraction, the Raven’s SPM, and then motivation questionnaires. General task instructions were delivered to the children as a group. Children were encouraged to ask clarifying questions, and the researcher responded with standardized additional instructions and ensured the child understood how to complete the task before proceeding with testing.

### Power Calculations and Sample Size

Calculations in G*Power (effect size *f*^2^=0.07; 80% power; α=.05) [50,51] indicated a sample size of 29 children per condition would be required to detect a small to moderate effect of each working memory training condition (adaptive, self-select, and stepwise) compared to the active control on the near transfer measures immediately postintervention (primary outcome). This effect size was based on previous research, which indicates working memory training programs produce a small to moderate effect compared to a control condition on near transfer measures [6,17]. The target sample size was increased to 32 children per condition to account for 10% attrition [22]. Our previous school-based trial of a working memory training program had a 65% child recruitment rate [22]. Thus, to achieve our target sample size, the participating primary school had to have at least 197 children enrolled in grades 2 to 5.

### Statistical Analysis

Primary analyses will be intention to treat (ITT) with sensitivity analyses only with children who completed the allocated intervention. Primary analyses will be separate linear regressions for each primary outcome at the primary end point (immediately postintervention) as the dependent variable, the outcome at baseline and the stratification factor (age) as covariates, and conditions (dummy coded with the active control as the reference) as the independent variables. Statistical significance will be set at α=.05 (2-tailed). If outcomes do not meet the normality assumption, a nonparametric bootstrap will be used. Effect sizes and 95% CI will be reported to demonstrate the magnitude of differences in outcomes between each training condition relative to the active control. Bayesian methods will be used to quantify the strength of evidence in favor of the null hypothesis and the alternative hypothesis. Interpretation of results will consider the strength of effects and *P* values. The same analytic approach will be applied to secondary outcomes and to both primary and secondary outcomes at the 6-month postintervention time point.

## Results

This trial was supported by internal research funding from the School of Psychological Sciences and the Turner Institute for Brain and Mental Health at Monash University, awarded in December 2019. Data collection was attempted in July 2021 but was ceased shortly after due to COVID-19 lockdowns in Melbourne. Data collection occurred from February 2022 to December 2022. Data are currently being processed and analyzed. It is anticipated that the results of this trial will be published by 2024.

## Discussion

The potential transfer of skills learned from a cognitive training intervention to other untrained activities is of interest to practitioners in health and education sectors, and families. Cognitive training interventions are intensive and typically comprise adaptive training activities, yet whether this approach to setting the difficulty of training activities maximizes transfer effects has only been systematically evaluated once in adults [7]. This trial aims to examine outcomes of working memory training using adaptive, self-select, and stepwise approaches to setting the difficulty level of training activities compared to an active control condition. This will be the first trial to systematically examine methods of setting the difficulty of working memory training activities in children. Our design includes evaluation of nonadaptive approaches (self-select and stepwise) and thereby eliminates task difficulty as a confounder, which was an important limitation of initial studies in the field.

This trial has important strengths. The trial design, methods, and analysis plan are in line with best-practice standards for cognitive training trials [1]. The trial was randomized, participants and assessors were blinded, and an active control condition was used. The active control condition (instead of a passive or no-contact control) was designed to be believable as an intervention, indistinguishable from the working memory training conditions in terms of the environment and motivating features, and thus was designed to ensure children experience a similar level of engagement, and experimenter and teacher attention to children allocated to the working memory training conditions. The set of outcome measures was designed to detect discrete levels of near transfer by minimizing confounders associated with training effects [4], and intermediate and far transfer effects are measured too. Training outcomes were assessed immediately postintervention and again at 6-month postintervention to determine any persisting training benefits. The trial is sufficiently powered to detect differences between the working memory training conditions and the active control condition. Importantly, our novel experimental working memory training interventions are designed specifically for this research and reflect the current understanding of the cognitive mechanisms of training: the processing demands of the training activities are (1) sufficiently unfamiliar such that the child needs to generate novel cognitive routines to do well, and the routines can then be applied to new activities that are similar but untrained; and (2) overlap with daily activities, such as following teacher directions, which overlap with daily activities. This is the first time the following instructions paradigm has been used as a working memory training activity. The nonadaptive approaches used in this trial reflect a similar approach to learning that children experience in daily activities, such as having autonomy over their learning (self-select), and mastering simpler concepts before complex concepts (stepwise). Motivation questionnaires will be used in analyses to determine if children in the training and active control conditions have similar motivation toward learning in the classroom and expectations of the intervention.

It is acknowledged that this trial was not powered to detect potential differences between the adaptive, self-select, and stepwise working memory training conditions. The lack of research and effect sizes from nonadaptive approaches makes calculating appropriate power challenging. We anticipate the differences in effect sizes between the 3 conditions will be small, and therefore a particularly large sample size would be required and this may not be practical or feasible.

This trial will determine whether the adaptive approach to setting the difficulty of working memory training activities is superior for maximizing training outcomes. It is our intention to present findings at national and international conferences and publish findings in peer-reviewed journal papers. Families and staff of the participating school will receive a summary of the findings. Findings will contribute to our understanding of the design of effective working memory training interventions, discrete levels of transfer of working memory training, and the malleability of cognitive functions in children. Knowledge gained could have implications for other types of cognitive training that target core domains such as attention and inhibitory control, and for other populations, including aging and dementia [3]. This work could help highlight the value of evidence-based principles of cognitive training and shift the focus from studying the effectiveness of different programs to more rigorous evaluations of the foundational principles of cognitive training. In summary, the results have the potential to influence the future direction and application of cognitive training.

## Abbreviations

ADHD-RS-5: ADHD-Rating Scale-5, home version
COMET: Common Assumptions of Cognitive Training
CONSORT: Consolidated Standards of Reporting Trials
IMI: Intrinsic Motivation Inventory
IMS: Intrinsic Motivation Scale
ITT: intention to treat
REDCap: Research Electronic Data Capture
SPM: Standard Progressive Matrices

## Data Availability

The data sets generated and analyzed during this study are not publicly available due to the restricted consent from participants but may be available from the corresponding author on reasonable request.
